# A scoping review of guidelines on caries management for children and young people to inform UK undergraduate core curriculum development

**DOI:** 10.1186/s12903-024-04278-7

**Published:** 2024-04-26

**Authors:** Faith Campbell, Helen Rogers, Rachel Goldsmith, Kathryn Rowles, Daniela Prócida Raggio, Nicola Innes

**Affiliations:** 1 Doctoral Fellow, University of Dundee, Dundee, UK; 2 Clinical Lecturer in Paediatric Dentistry, School of Dental Sciences, Newcastle University, Newcastle, UK; 3 Specialty Registrar in Paediatric Dentistry, Newcastle Dental Hospital, Newcastle, UK; 4 Clinical Lecturer in Paediatric Dentistry, Cardiff University, Cardiff, UK; 5 Department of Paediatric Dentistry, School of Dentistry, University of São Paulo, São Paulo, Brazil; 6 Paediatric Dentistry, Honorary Consultant and Head of Cardiff Dental School, Cardiff University, Cardiff, UK

**Keywords:** Dental education, Paediatric dentistry, Cariology, Dental caries

## Abstract

**Background:**

Current evidence in cariology teaching is not consistently reflected in paediatric dentistry in the United Kingdom (UK). Many dental schools are not consistently teaching biological approaches to caries management, with outdated or complex methods being taught outwith the purview of general dental practitioners. This scoping review aimed to map current guidelines on the management of caries in children and young people. This is part of a work package to inform the consensus and development of a UK-wide caries management curriculum for paediatric dentistry.

**Methods:**

A search of electronic databases for peer reviewed literature was performed using Cochrane Library, MEDLINE via PubMed, TRIP Medical Database and Web of Science. Hand searching was undertaken for grey literature (citations of sources of evidence, websites of global organisations and Google Web Search™ (Google LLC, California, USA). Results from databases were screened independently, concurrently by two reviewers. Full texts were obtained, and reviewers met to discuss any disagreement for both database and hand searching.

**Results:**

This review identified 16 guidelines suitable for inclusion. After quality appraisal, eight were selected for synthesis and interpretation. Key themes included the shift towards selective caries removal and avoidance of complete caries removal unless in specific circumstances in anterior teeth. For “early lesions” in primary and permanent teeth with and without cavitation, several guidelines recommend biological management including site specific prevention and fissure sealants.

**Conclusions:**

This review mapping current cariology guidelines for children and young people found gaps in the literature including classification of early carious lesions and management of early cavitated lesions. Areas identified for further exploration include integration of biological caries management into treatment planning, selective caries removal and whether pulpotomy is specialist-level treatment, requiring referral. These results will inform consensus recommendations in the UK, using Delphi methods.

**Supplementary Information:**

The online version contains supplementary material available at 10.1186/s12903-024-04278-7.

## Introduction

### Background

Concepts on the management of caries have shifted significantly over the past twenty years, with the evidence base demonstrating the efficacy and/or effectiveness, and benefits of minimally invasive dentistry (MID) [1]. Change in practice does not happen through production of evidence, but through its implementation and it can be difficult to change practitioners’ ways of working once these are established. One of the biggest opportunities to effect change in professional practice is through the undergraduate dental education of future clinicians [2]. However, the change in evidence towards MID was not reflected in the findings of a recent national survey, which found wide disparity in the content and methods of teaching caries management in children and young people (CYP) to undergraduate dental students in the UK [3]. There was wide variation in paediatric caries management methods taught to the next generation of dental practitioners, with outdated practice still evident in teaching. This impacts on the appropriateness of care provided for CYP’s oral and dental health and emphasises the need for recommendations to support a national curriculum for the management of caries in CYP. Within this work we defined children and young people as those under the age of 18, this is the definition used by the UK government, United Nations Convention on the Rights of the Child and civil legislation in England and Wales [4].

### Rationale for the review

There are a number of guidelines, produced by various organisations internationally, on the management of dental caries [5, 6]. Within paediatric dentistry, there are several international groups producing such guidelines for professional bodies including the International-, American-, European- and British Associations for Paediatric Dentistry and others within the UK alone, such as the Scottish Dental Clinical Effectiveness Programme and the Scottish Intercollegiate Guidelines Network [5, 7–18]. However, these are of variable quality and each are designed for specific environments. High-quality guidelines that are UK-relevant should be informing education and practice within the UK and could be used for the development of recommendations for a core curriculum. To begin development of these, we aimed to map the recommendations from current guidelines through a scoping review. Scoping reviews can be defined as “a type of evidence synthesis that aims to systematically identify and map the breadth of evidence available on a particular topic, field, concept, or issue, often irrespective of source (i.e., primary research, reviews, non-empirical evidence) within or across particular contexts” [4].

A preliminary search of MEDLINE, the Cochrane Database of Systematic Reviews and JBI Evidence Synthesis found no current or historic systematic reviews or scoping reviews on this topic. This evaluation of the current guidance on the management of caries in CYP forms part of a package of work to inform the development of a position statement and nationally agreed curricula on caries management teaching for undergraduate students within UK dental schools.

This scoping review identifies and appraises the quality of clinical guidelines relevant to the management of caries in CYP and maps their recommendations. This will inform the development of a consensus on the curricula for teaching caries management to undergraduate Dentistry and Dental Hygiene and Therapy students at UK dental schools.

### Aim and objectives

The aim of the review was to evaluate current guidelines for caries management in CYP to inform undergraduate dental education in the UK.

The specific objectives were to:

1. Identify guidelines relevant to the management of caries in CYP;
2. Appraise the quality of the guidelines using the AGREE II tool;
3. Synthesise recommendations from relevant guidelines of acceptable quality to guide undergraduate teaching of caries management in CYP in the UK; and
4. Identify gaps in the current guidelines regarding the management of caries in CYP.

## Methods

The protocol for this scoping review was registered prospectively on 27/03/23 on Open Science Framework (10.17605/OSF.IO/SBHC3). The review was reported according to PRISMA-ScR (see supplementary material) [19].

### Eligibility

#### Inclusion criteria

To be included, the publication must:

- Be a clinical guideline;
- Contain information on the management of dental caries in CYP;
- Have been developed using a structured guideline methodology;
- Provide recommendations on the management of dental caries in children and/or young people;
- Be endorsed or created by a recognised dental organisation;
- Be written by multiple authors; and,
- Be published from 2007 onwards.

Guidelines from any country, relating to any dental setting (primary, secondary, or tertiary care) and written in English (these were considered likely to be most relevant to the UK setting) were considered. Studies published since 2007 were included as this was when the first clinical trial of the Hall Technique was published [20]. This is generally considered a time when an institutional shift in the thinking behind caries management, towards biological management of caries, began to occur.

The definition of, and ages at which people are considered to be, children and young people vary internationally therefore any publication that used the term children and or young people was included to ensure relevant publications were not excluded based on this point.

#### Exclusion criteria

Expert opinion papers, position statements and guidelines produced by industry were not considered for inclusion.

### Types of sources

Only guidelines, or conference proceedings subsequently published as guidelines, endorsed or created by recognised dental organisations were considered.

### Selection of sources of evidence

The search strategy aimed to locate both published and unpublished guidelines. An initial limited search of MEDLINE via PubMed was undertaken to identify articles on the topic. The text words contained in the titles and abstracts of relevant articles, and the index terms used to describe the articles were used to develop a comprehensive search strategy (Appendix 1). This search strategy, including all identified keywords and index terms, was adapted for each included database and information source.

The databases searched included Cochrane Library, MEDLINE via PubMed, TRIP Medical Database and Web of Science. Sources of unpublished guidelines/grey literature were searched by contacting authors of existing guidelines to find out whether they were aware of any others underway. Webpages of major dental organisations in this field known to the authors were also searched, alongside a hand search of conference proceedings and a Google Web Search™ (Google LLC, California, United States of America). The reference lists of all included sources of evidence were screened for additional studies.

### Search strategy

**Fig. 1:**
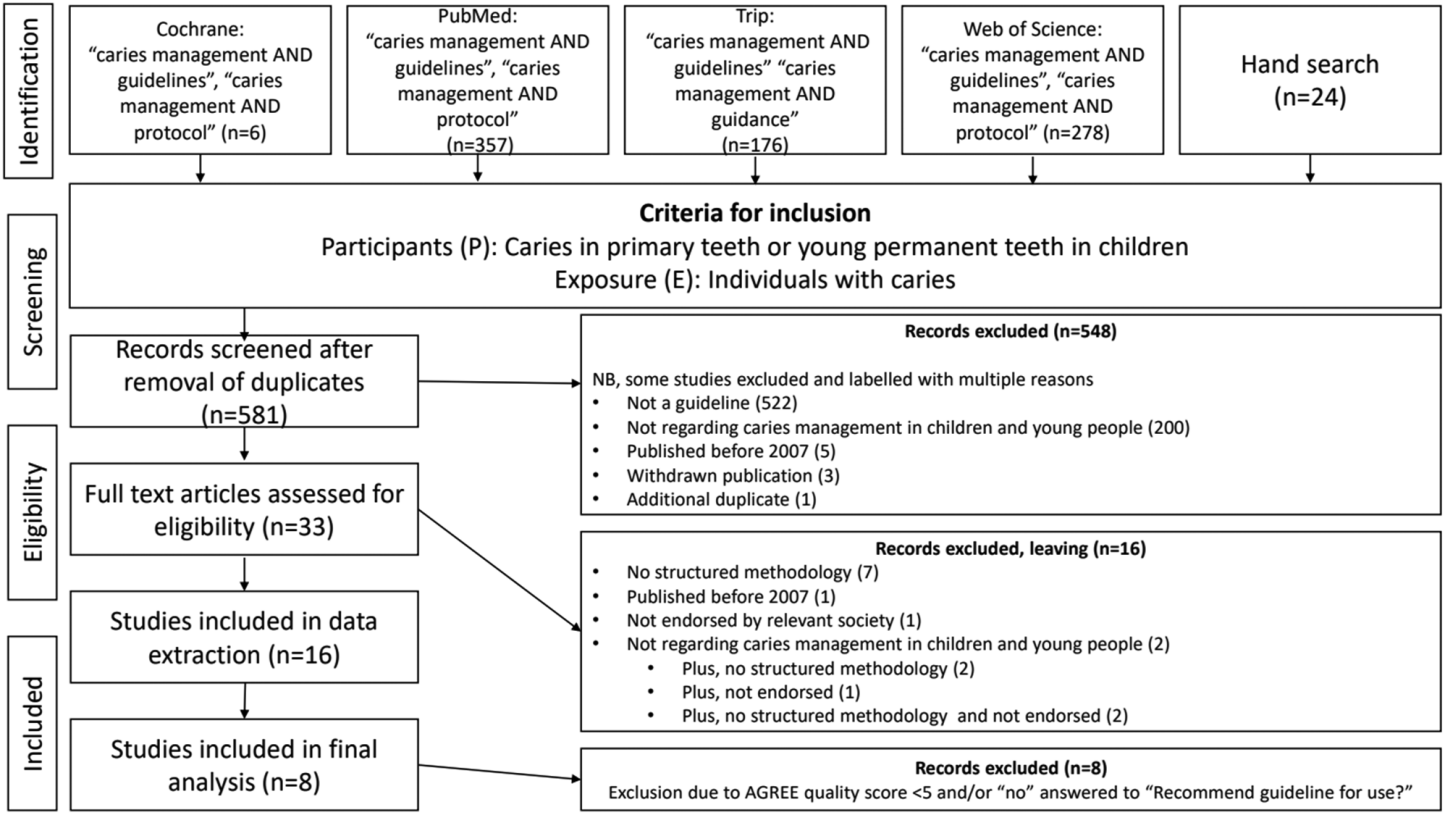
Flow diagram of databases searched, findings and outputs

The search (Fig. 1) was conducted for guidelines published between 01/01/2007 and 21/04/2023. The TRIP database search was further modified to include “guidance” as “guidelines” yielded only five results. The search was repeated on 25/01/2024 and no new guidelines were identified that met the criteria for this review.

### Selection of guidelines

Results from databases were screened independently and concurrently by two reviewers (FC and NI) against the inclusion criteria using Rayyan© software (Rayyan, Massachusetts, United States of America) [21]. Hand searching was conducted by one researcher (FC). All findings were compiled into a Microsoft® Excel® (Microsoft® Corporation, Washington, United States of America) spreadsheet (Appendix 2). Full texts were obtained, and reviewers met to discuss any disagreement for both database and hand searching.

Guideline selection was guided by the minimum score of 4.5 in the overall AGREE II scoring indicating quality standard (Table 1), but reviewers also included wider considerations relating to the relevance to the UK education and wider paediatric dentistry environment as well as the paediatric caries-specific curriculum.

**Table 1:** Domains covered by the AGREE II quality appraisal tool (AGREE II)

Checklist item and description	Overall focus	Questions to reviewers
Domain 1. Scope and purpose	The overall aim of the guideline, the specific health questions, and the target population	1. The overall objective(s) of the guideline is (are) specifically described 2. The health question(s) covered by the guideline is (are) specifically described 3. The population (patients, public, etc.) to whom the guideline is meant to apply is specifically described
Domain 2. Stakeholder Involvement	Focuses on the extent to which the guideline was developed by the appropriate stakeholders and represents the views of its intended users	4. The guideline development group includes individuals from all relevant professional groups 5. The views and preferences of the target population (patients, public, etc.) have been sought. 6. The target users of the guideline are clearly defined
Domain 3. Rigour of Development	Relates to the process used to gather and synthesise the evidence, the methods to formulate the recommendations, and to update them	7. Systematic methods were used to search for evidence 8. The criteria for selecting the evidence are clearly described 9. The strengths and limitations of the body of evidence are clearly described 10. The methods for formulating the recommendations are clearly described 11. The health benefits, side effects, and risks have been considered in formulating the recommendations 12. There is an explicit link between the recommendations and the supporting evidence 13. The guideline has been externally reviewed by experts prior to its publication 14. A procedure for updating the guideline is provided
Domain 4. Clarity of Presentation	Deals with the language, structure, and format of the guideline	15. The recommendations are specific and unambiguous 16. The different options for management of the condition or health issue are clearly presented 17. Key recommendations are easily identifiable
Domain 5. Applicability	Pertains to the likely barriers and facilitators to implementation, strategies to improve uptake, and resource implications of applying the guideline	18. The guideline describes facilitators and barriers to its application 19. The guideline provides advice and/or tools on how the recommendations can be put into practice 20. The potential resource implications of applying the recommendations have been considered 21. The guideline presents monitoring and/or auditing criteria
Domain 6. Editorial Independence	Concerned with the formulation of recommendations not being unduly biased with competing interests	22. The views of the funding body have not influenced the content of the guideline 23. Competing interests of guideline development group members have been recorded and addressed
Overall assessment	Includes the rating of the overall quality of the guideline and whether the guideline would be recommended for use in practice.	Would you recommend this guideline for use in practice?

### Data extraction and charting

Guidelines initially considered to meet the inclusion criteria were distributed between two teams of three reviewers for independent, duplicate data extraction (calibrated through data extraction and discussion of one guideline) with discussion to achieve a single agreed dataset. Microsoft® Forms® (Microsoft® Corporation, Washington, United States of America) was used for data extraction (Appendix 3) and quality appraisal.

### Quality appraisal

Quality appraisal was undertaken by each reviewer within the same two teams alongside data extraction using the AGREE II criteria [22]. Table 1 details the AGREE II tool and the domains covered. Calibration was undertaken alongside calibration for data extraction. Reviewers were blinded and quality appraisal was undertaken using Microsoft Forms® (Microsoft® Corporation, Washington, United States of America). Any disagreements were discussed, and a consensus reached for each domain.

### Synthesis of results

Results were collated by one reviewer in Microsoft® Excel® (Microsoft® Corporation, Washington, United States of America). Reviewers met to discuss any conflicts and agree the final dataset.

Data from each guideline were tabulated and summarised in categories relating to the specific area of caries management including depth of lesion and primary/permanent dentition.

## Results

The results of the search, screening and agreement for guidelines published between 1/1/07 and 25/01/2024 is shown in Fig. 1.

Of the 581 guidelines identified from the search, there were 16 guidelines meeting the eligibility and quality criteria for inclusion. Table 2 shows the characteristics of the guidelines and Table 3 the quality was appraised (Table 3).

**Table 2:** Characteristics of the identified guidelines that met the eligibility criteria for this scoping review and were taken forward for narrative synthesis (study demographics)

Guideline ID	Name and citation	Year of publication	Guideline development organisation	Country	Guideline panel
1	Evidence-based clinical practice guideline for the use of pit-and-fissure sealants [27]	2016	AAPD^1^	USA	Individuals recognised for their level of clinical and research expertise and who represented the different perspectives required for clinical decision making (general dentists, paediatric dentists, dental hygienists, and health policy makers). Methodologists from the American Dental Association Center for Evidence-Based Dentistry oversaw the guideline development process.
2	Use of vital pulp therapies in primary teeth with deep caries lesions [17]	2017	AAPD^1^	USA	Workgroup and stakeholders: panel consisted of pediatric dentists in public and private practice involved in research and education; the stakeholders consisted of authors of the systematic review in addition to representatives from general dentistry, governmental and non-governmental agencies, and international and specialty dental organisations. External stakeholders. External and internal stakeholders reviewed the document periodically during the process of development of the guideline. Stakeholders also participated in anonymous surveys to determine the scope and outcomes of the guideline.
3	Use of silver diamine fluoride for dental caries management in children and adolescents, including those with special health care needs [16]	2017	AAPD^1^	USA	As for 2
4	Prevention and management of dental caries in children [5]	2018	SDCEP^2^	Scotland, UK	Individuals from a range of relevant branches of the dental profession and two patient representatives.
5	Use of non-vital pulp therapies in primary teeth [10]	2020	AAPD^1^	USA	Seven paediatric dentists in public and private practice involved in research and education; the stakeholders consisted of representatives from general dentistry, governmental and nongovernmental agencies, and international and specialty dental organisations. External and internal stakeholders reviewed the document during the process of development of the guideline. Internal stakeholders also participated in anonymous surveys to determine the scope and outcomes of the guideline. All stakeholder comments were considered and addressed in the work group meetings.
6	Pulp therapy for primary and immature permanent teeth [12]	2020	AAPD^1^	USA	Reiteration of previous guidance developed by AAPD Council on Clinical Affairs in 1991. Not accessible for review of panel members and no indication of who consisted of the review panel. American Academy of Pediatric Dentistry. Pulp therapy for primary and young permanent teeth. In: American Academy of Pediatric Dentistry Reference Manual 1991–1992. Chicago, Ill.: American Academy of Pediatric Dentistry; 1991:53 − 7
7	Paediatric restorative dentistry [14]	2022	AAPD^1^	USA	As for 6, council of clinical affairs developed original guidance in 1991. American Academy of Pediatric Dentistry. Guidelines for pediatric restorative dentistry 1991. In: American Academy of Pediatric Dentistry Reference Manual 1991–1992. Chicago, Ill.: American Academy of Pediatric Dentistry; 1991:57 − 9
8	Caries-risk assessment and management for infants, children, and adolescents [35]	2022	AAPD^1^	USA	As for 6, council of clinical affairs developed original guidance in 2002. American Academy of Pediatric Dentistry. The use of a caries-risk assessment tool (CAT) for infants, children, and adolescents. Pediatr Dent 2002;24 [7]:15 − 7.

**Table 3:** Summary of guideline recommendations and AGREE II scores (*n* = 16 guidelines)

Guideline ID	Guideline development organisation	Guideline name	Contains recommendations on management of						Endorse preventive resin restorations?	Mention/ endorse silver diamine fluoride (SDF)?	Endorse therapeutic sealants?	Mean AGREE II score (overall quality) from a maximum of 7 indicating highest quality	Recommend guideline for use?
			Primary teeth			Permanent teeth							
			Early carious lesions	Deep carious lesions (> 1/3 into dentine)	Carious lesions into pulp	Early carious lesions	Deep carious lesions (> 1/3 into dentine)	Carious lesions into pulp					
1	AAPD	Evidence-based Clinical Practice Guideline for the Use of Pit-and-Fissure Sealants	Yes	No	No	Yes	No	No	Not discussed	No	Yes	**6.5**	Yes
2	AAPD	Use of Vital Pulp Therapies in Primary Teeth with Deep Caries Lesions	No	Yes	Yes	No	No	No	Not discussed	No	Not discussed	**5**	Yes
3	AAPD	Use of Silver Diamine Fluoride for Dental Caries Management in Children and Adolescents, Including Those with Special Health Care Needs	No	Yes (unclear on depth)	No	No	No	No	Not discussed	Yes/ Yes	Not discussed	**5**	Yes
4	SDCEP	Prevention and Management of Dental Caries in Children	Yes	Yes	Yes	Yes	Yes	Yes	Not discussed	No	Yes	**7**	Yes
5	AAPD	Use of Non-Vital Pulp Therapies in Primary Teeth	No	No	Yes	No	No	No	Not discussed	No	Not discussed	**6**	Yes
6	AAPD	Pulp Therapy for Primary and Immature Permanent Teeth	No	Yes	Yes	No	Yes	Yes	Not discussed/ No	No	Not discussed	**4.5**	Yes
7	AAPD	Pediatric Restorative Dentistry	Yes	Yes	Yes	Yes	Yes	No	No	Yes/ Yes	Not discussed	**5**	Yes
8	AAPD	Caries-Risk Assessment and Management for Infants, Children, and Adolescents	Yes	Yes	No	No	No	No	Not discussed	Yes/Yes	Not discussed	2.6*	Yes
9	EAPD	Best clinical practice guidance for treating deep carious lesions in primary teeth: an EAPD policy document	No	Yes	Yes	No	No	No	No	Yes/Yes	No	3	No
10	IAPD	Management of Early Childhood Caries: Foundational Articles and Recommendations	Yes	Yes	No	No	No	No	Not discussed	Yes/Yes	Yes	2	No
11	IAPD	Pulp Therapy for Primary and Young Permanent Teeth: Foundational Articles and Recommendations	No	No	Yes	No	No	Yes	No	No	Not discussed	1.5	No
12	IAPD	Hall Technique for Crown Placement in Primary Molars: Foundational Articles and Recommendations	Yes	Yes	No	No	No	No	Not discussed	No	Not discussed	1.5	No
13	IAPD	Atraumatic Restorative Treatment: Foundational Articles and Recommendations	Yes	Yes	Yes	Yes	Yes	Yes	Not discussed	No	Not discussed	1	No
14	IAPD	Minimal Invasive Dentistry: Foundations Articles and consensus recommendations	Unclear	Unclear	No	Unclear	Unclear	No	Not discussed	Yes	Not discussed	1	No
16	IAPD	Management of severe early childhood caries	Yes	Yes	Yes	No	No	No	No	Not discussed	No	6**	No

*Legend* *Although the AGREE II scores for this guideline were low, after independent review by a third person and discussion within the whole research team it was decided that it should be included. There were limited recommendations that were relevant which is why some reviewers had given it a low score, however the team concluded that the few recommendations that were relevant were of acceptable quality. ** Although the AGREE II scores for this guideline were high, independent review and consideration of the wider content of the guideline, it was not taken further for narrative review. This was due to the limited number of recommendations and the evidence base for this. The reviewers did feel that the guideline was presented in a methodical and clear manner hence the high AGREE II score however when evaluating the most important aspects of quality, the guideline was deemed inadequate. **Bold type indicates** meeting the AGREE II threshold for inclusion

There were eight guidelines meeting the set quality standard and considered appropriate for inclusion, for which data extraction and synthesis were carried out (Tables 4 and 5).

**Table 4:** Synthesis of the guideline recommendations for primary teeth on management of carious lesions

Treatment option		Recommendation	Guideline(s) that the recommendation is derived from
Caries-related	Restoration-related		
**Management of early carious lesions in primary teeth**			
No caries removal	Fissure sealants	For sound occlusal surfaces and early carious lesions [5, 27]	SDCEP [5] AAPD Pit and Fissure sealants [27]
No caries removal	Resin infiltration	Consider for small non-cavitated lesions [5, 14]	AAPD Paediatric Restorative Dentistry [14] SDCEP [5]
Site specific prevention	No restoration required	For occlusal and interproximal caries, intervene if the lesion is progressing (transitioning) [5, 14]	SDCEP [5] AAPD Pediatric Restorative Dentistry [14]
Active surveillance	No restoration required	Non-cavitated (white spot) caries lesions [14]	AAPD, Pediatric Restorative Dentistry [14]
**Management of deep carious lesions > 1/3 into dentine in primary teeth**			
No or selective caries removal	Preformed metal crowns (PMC)	Multi-surface lesions on posterior teeth: A preformed metal crown is the restoration of choice (with SDCEP advocating the Hall technique) [5, 14].	SDCEP [5] AAPD (Pediatric Restorative Dentistry) [14]
		Preformed metal crowns indicated for high-risk children with large or multi-surface cavitated or non-cavitated lesions on primary molars. (General recommendation for guideline is selective caries removal) [14].	AAPD (Pediatric Restorative Dentistry) [14]
	Resin-based composites	Resin-based composites can be used as Class I and Class II restorations in primary and permanent molars [14].	(AAPD, Pediatric Restorative Dentistry) [14]
Selective caries removal	PMC for proximal lesions PMC or plastic restoration for occlusal lesions	For teeth with a healthy pulp and no pulpal exposure, reversible pulpitis or when complete caries removal is likely to result in pulp exposure [5, 14, 27].	SDCEP [5] AAPD (Pediatric Restorative Dentistry) [14] AAPD (Pit and fissure sealants) [27]
		For teeth with advanced occlusal caries incomplete caries removal with restoration using plastic restoration [5].	SDCEP [5]
		It is essential to ensure a good seal with a permanent restoration A preformed metal crown is the material of choice for interproximal cavities [5].	SDCEP [5]
	Indirect pulp therapy	Indicated for primary teeth with deep caries, with success being independent of the type of medicament used, therefore this should be dictated by clinician preference [14].	AAPD (Paediatric Restorative Dentistry) [14]
Complete caries removal	No material recommended	Only advocated for anterior teeth and if a child cannot co-operate with caries removal [5].	SDCEP [5]
Silver diamine fluoride (SDF)	No restoration mentioned	Advocated for: • High caries-risk patients with anterior or posterior active cavitated lesions • Cavitated caries lesions in individuals presenting with behavioural or medical management challenges • Patients with multiple cavitated caries lesions that may not all be treated in one visit • Difficult to treat cavitated dental caries lesions • Patients without access to or with difficulty accessing dental care • Active cavitated caries lesions with no clinical signs of pulp involvement. • Teeth with deep caries lesions should be closely monitored clinically and radiographically [16, 34]	AAPD, SDF [16] EAPD [34]
Non-restorative cavity control	No restoration required	Can be considered if selective caries removal and conventional restoration or preformed metal crown placement using the Hall technique is not suitable or the tooth is unrestorable [5].	SDCEP [5]
Atraumatic restorative treatment (ART). NB sometimes referred to as intermediate therapeutic restoration (ITR) by AAPD guidelines	No specific restorative material mentioned	For children at moderate and high risk of caries, cavitated or enlarging carious lesions should be restored [5]. ART is appropriate for single surface cavities but not multi-surface [5]. For large symptomatic carious lesions: Incomplete caries removal, dressing with glass ionomer cement and review symptoms in three to seven days [35]. ITR may be used until permanent restorations can be placed [35].	SDCEP [5] AAPD (Caries Risk Assessment and Management) [35]
**Management of caries into pulp in primary teeth**			
SDF		SDF is **not** recommended for carious lesions into pulp in primary teeth [16].	AAPD (SDF) [16]
Selective caries removal	No restoration type mentioned	Consider for deep caries and normal pulp status or reversible pulpitis when complete caries removal is likely to result in pulp exposure [14].	AAPD (Paediatric Restorative Dentistry) [14]
	PMC (Hall Technique)	Place a crown using the Hall Technique or if an occlusal lesion, carry out selective caries removal, avoiding the pulp, and restore using composite, resin modified glass ionomer, compomer or glass ionomer [5].	SDCEP [5]
	Radiopaque liner and restoration with a material that completely seals the dentine from the oral environment	For deep carious lesions without evidence of periradicular pathology a radiopaque liner such as a dentine bonding agent, resin modified glass ionomer, calcium hydroxide, or mineral trioxide aggregate (MTA) (or any other biocompatible material) is placed over the remaining carious dentin to stimulate healing and repair [12]. The liner that is placed over the dentin (calcium hydroxide, glass ionomer, or bonding agents) does not affect the indirect pulp therapy (IPT) success. The tooth then is restored with a material that seals the tooth from microleakage [12].	AAPD Pulp Therapy for Primary and Permanent teeth [12]
	ART NB – this is referred to as ITR (Intermediate Therapeutic Restoration) in AAPD Guideline	**In the presence of symptoms** When there are signs of reversible pulpitis and using glass ionomer cement for caries control, current literature indicates there is no conclusive evidence that it is necessary to re-enter the tooth to remove the residual caries [12]. Where there are symptoms of pain that may be due to food packing or pulpitis with reversible symptoms, but the diagnosis is uncertain, a temporary dressing can be placed into the cavity and the patient reviewed 3–7 days later to check symptoms. Resolution of the symptoms at review will indicate that the pulpitis was reversible, and a Hall crown or suitable restoration can then be placed [5]. If symptoms do not resolve or worsen then extraction or pulpotomy should be considered [5]. If the tooth is close to exfoliation, consider applying a dressing. When deciding whether to undertake a pulpotomy or extract a tooth; If the child is anxious, and/or it is their first visit, gently remove gross debris from the cavity, and apply corticosteroid antibiotic paste under a temporary dressing. Ideally, if cooperation permits, open the pulp chamber under local anaesthesia and apply corticosteroid paste directly to the pulp, then place a dressing. Prescribe pain relief then carry out a pulpotomy or extract the tooth at a later date [5].	AAPD (Pulp therapy for primary and immature permanent teeth) [12] SDCEP [5]
		For multi-surface lesions, a stainless-steel crown is the restoration of choice. Amalgam or composite resin can provide a functional alternative when the primary tooth has a life span of two years or less [12].	(AAPD, Pulp therapy for primary and immature permanent teeth) [12]
	Biocompatible material	Direct pulp caps are recommended using a biocompatible material or MTA [12, 14]. Bioactive materials can be used for remineralisation and pulp capping [14].	AAPD (Pulp therapy for primary and immature permanent teeth) [12], AAPD (Paediatric Restorative Dentistry) [14]
Pulp Treatment			
Pulpotomy		Use MTA, formocresol, and tricalcium silicate in vital primary teeth with deep caries lesions treated with pulpotomy due to pulp exposure during carious dentin removal with the ultimate decision being clinical preference. Do not use calcium hydroxide (CaOH) in vital primary teeth with deep caries lesions treated with pulpotomy due to pulp exposure during carious dentin removal [17]. Where a radiograph shows no clear separation between the carious lesion and the dental pulp, it is likely that the carious lesion has encroached significantly on the dental pulp and a pulpotomy will be necessary. For a child in pain due to pulpitis in a vital primary tooth with irreversible symptoms and no evidence of dental abscess, consider carrying out a pulpotomy to preserve the tooth and to avoid the need for an extraction. If the child is cooperative, extract the tooth, even if the infection is asymptomatic [5].	AAPD (Use of vital pulp therapies in primary teeth with deep caries lesions) [17] SDCEP [5]
Pulpectomy		Pulpectomy should be considered for non-vital primary teeth without preoperative root resorption and should be considered as preferable compared to lesion sterilisation tissue repair (LSTR) [10]. In exceptional circumstances if the tooth is restorable, consider a pulpectomy, which may require referral [5]. In some cases, local measures to bring infection under control may be appropriate. If the child is uncooperative refer to a specialist for treatment [5].	AAPD (Use of Non-Vital Pulp Therapies in Primary Teeth) [10] SDCEP [5]

**Table 5:** Synthesis of guideline recommendations for permanent teeth

Treatment option		Recommendation	Guideline(s) that the recommendation is derived from
Caries-related	Restoration-related		
**Management of early carious lesions in permanent teeth**			
No caries removal	Site specific prevention	For proximal lesions; Identify and arrest early enamel-only lesions paying particular attention to the mesial surface of first permanent molars. Carry out site specific prevention and monitor with bitewing radiographs. Ensure that the parent/carer is fully aware of the potential impact on their child’s oral health [5].	SDCEP [5]
No caries removal	Fissure sealants	Fissure sealants should be used for both sound occlusal surfaces and early carious lesions [27]. For occlusal caries (or proximal lesions where site specific prevention is not suitable) place a resin fissure sealant [5]. If early occlusal dentinal caries is inadvertently sealed in, provided the sealant is maintained, the caries is unlikely to progress [5]. Clinically review sealant for wear and check integrity at every recall visit physically with a probe. If the sealant is worn, top it up [5]. If the sealant is not adherent to the tooth, remove it and replace. If the lesion has progressed, adopt an alternative management strategy [5]. Radiographically review in line with current recommendations [5]. If the tooth is only partially erupted, or the child’s cooperation is insufficient for placement of a resin fissure sealant or a restoration, consider the use of a glass ionomer material as a temporary sealant or restoration [5].	AAPD (Pit and fissure sealants) [27] SDCEP [5]
No caries removal	Resin infiltration	Resin infiltration is indicated as an adjunct to preventive measures for primary and permanent teeth with small, noncavitated interproximal caries lesions to reduce lesion progression and for white-spot lesions to improve their clinical appearance [14].	AAPD (Pediatric Restorative Dentistry) [14]
Glass Ionomer Cement (GIC)		Do not use GIC; Evidence is insufficient to support the use of conventional or resin modified GIC (RMGIC) as a long-term restorative material in permanent teeth [14].	AAPD (Pediatric Restorative Dentistry) [14]
**Management of deep carious lesions > 1/3 into dentine in permanent teeth**			
Selective caries removal	No material specified	For moderate occlusal and proximal dentinal caries - Carry out selective caries removal or, if necessary to allow sufficient depth and surface area for the restorative material, carry out complete caries removal prior to restoration, seal the remaining fissures [5]. For extensive occlusal and proximal dentinal caries - Carry out stepwise caries removal, temporise with an obvious temporary material and restore with a permanent restoration after 6 to 12 months. Seal the remaining fissures [5]. For a healthy pulp, where there is no pulpal exposure consider; protective liners (liner over floor of cavity when no exposure and all caries removed) or indirect pulp treatment by leaving caries over floor of cavity then placement of biocompatible liner for biological seal. The tooth should then be restored with a material that seals the tooth from microleakage [12]. Incomplete caries removal should be considered in primary and permanent teeth with deep caries and normal pulp status or reversible pulpitis when complete caries removal is likely to result in pulp exposure [12]. Do not use GIC as a long-term restorative material [14].	SDCEP [5] SDCEP [5] AAPD (Pulp therapy for primary and immature permanent teeth) [12] AAPD (Pulp therapy for primary and immature permanent teeth) [12] AAPD (Pediatric Restorative Dentistry) [14]
	Atraumatic Restorative Technique. NB referred to as ITR in AAPD guidelines.	ART (ITR) using high-viscosity glass-ionomer cements may be used as single surface temporary restoration for both primary and permanent teeth [14]. Additionally, ITR may be used for caries control in children with multiple open caries lesions, prior to definitive restoration of the teeth. Evidence is insufficient to support the use of conventional or RMGICs as long-term restorative material in permanent teeth [14].	AAPD (Pediatric Restorative Dentistry) [14]
	Indirect pulp therapy	In the presence of signs of irreversible pulpitis; ITR with glass ionomer cements may be used for caries control. Current literature indicates there is no conclusive evidence that it is necessary to re-enter the tooth to remove the residual caries. Indirect pulp therapy (IPT) including selective and stepwise caries removal; leave caries, line and don’t re-enter [12].	AAPD (Pulp therapy for primary and immature permanent teeth) [12]
Use of bioactive materials		Bioactive materials can be used for remineralisation and pulp capping [14].	AAPD (Pediatric Restorative Dentistry) [14]
Complete caries removal (no material mentioned)		For permanent anterior teeth with advanced caries - Completely remove caries and restore or consider selective caries removal and restore [5].	SDCEP [5]
**Management of caries into pulp in permanent teeth**			
Selective caries removal	No restorative material specified	In the presence of reversible pulpitis carry out stepwise or complete caries removal, taking care to avoid the pulp, and place a restoration. It may be necessary to provide a temporary dressing and review the tooth before placing a permanent restoration later (stepwise only as if complete then not deep enough to be into pulp) [5].	SDCEP [5]
	Pulp caps	Bioactive materials can be used for remineralisation and pulp capping [14].	AAPD (Paediatric Restorative Dentistry) [14]
		Selective caries removal as a pulp cap: In the presence of signs of irreversible pulpitis ITR with glass ionomer cements may be used for caries control. Current literature indicates there is no conclusive evidence that it is necessary to re-enter the tooth to remove the residual caries. IPT including selective and stepwise caries removal; leave caries, line and don’t re-enter [12].	AAPD (Pulp therapy for primary and immature permanent teeth) [12]
		For small carious pulpal exposures place a direct pulp cap [12].	AAPD (Pulp therapy for primary and immature permanent teeth) [12]
Pulp Therapy			
Partial pulpotomy		For larger exposures partial pulpotomy is indicated in a young permanent tooth, for a carious pulp exposure in which the pulp bleeding is controlled within several minutes. The tooth must be vital, with a diagnosis of normal pulp or reversible pulpitis. Use either CaOH or MTA [12].	AAPD (Pulp therapy for primary and immature permanent teeth) [12]
Full pulpotomy		A full pulpotomy is indicated in immature permanent teeth with carious pulpal exposure as an interim procedure to allow continued root development (apexogenesis). It also may be performed as an emergency procedure for temporary relief of symptoms until a definitive root canal treatment can be accomplished. Indications for apexification: non-vital permanent teeth with incompletely formed roots [12].	AAPD (Pulp therapy for primary and immature permanent teeth) [12]
Endodontic treatment – root canal therapy (Pulpectomy)		In the presence of irreversible pulpitis or dental abscess/periradicular periodontitis either carry out a root canal therapy or extract the tooth. To relieve symptoms, and to allow time for long term treatment planning, consider root canal therapy and dressing of the root canals, before deciding on extraction of a permanent tooth [5].	SDCEP [5]
		Indications: a restorable permanent tooth with a closed apex that exhibits irreversible pulpitis or a necrotic pulp. For root canal-treated teeth with unresolved peri-radicular lesions, root canals that are not accessible from the conventional coronal approach, or calcification of the root canal space, endodontic treatment of a more specialised nature may be indicated [12].	AAPD (Pulp therapy for primary and immature permanent teeth) [12]
Other treatment			
Regenerative endodontic technique		Indications: nonvital permanent teeth with incompletely formed roots [12].	AAPD (Pulp therapy for primary and immature permanent teeth) [12]
Extraction		In the presence of irreversible pulpitis or dental abscess/periradicular periodontitis either carry out a root canal therapy or extract the tooth. If the tooth is unrestorable, extract the tooth and try to avoid extractions at a child’s first visit if at all possible [5].	SDCEP [5]
Temporisation		If the tooth is unrestorable and child is unable to cope with the extraction (due to a learning disability or where behaviour management techniques have been unsuccessful), temporise the tooth, continue prevention and refer the child for specialist paediatric dental or orthodontic opinion [5].	SDCEP [5]

## Discussion

Based on guideline quality indicators and relevance to education on the management of dental caries in CYP within the UK setting, eight guidelines were selected for synthesis of their clinical recommendations. The review was carried out to provide an evidence-base to inform the development of a consensus for the undergraduate curriculum for caries management in CYP, specific to the UK. The need for this consensus was highlighted by a UK survey evaluating current teaching practices for caries management in children and young adults, which showed great variance in the content of teaching and a delay in modernising curricula to keep up with best available evidence [3].

Initial screening identified 16 guidelines but following quality appraisal using the AGREE II tool, only eight were suitable for inclusion in the data synthesis. The exclusion of nine of the 16 guidelines demonstrates the constant problem with quality of evidence and waste [23]. In this case, the quality issues surrounded the development and reporting of guidelines. One of the most common reasons for exclusion of guidelines from synthesis in this study related to the lack of detail and transparency around the process for development of the guidelines, meaning that quality for inclusion could not be adequately determined. These guidelines did not have listed authors to contact to clarify this for inclusion.

### Biological caries management approaches

Preformed metal crowns are recommended in all guidance for the restoration of multi-surface carious lesions. However, in UK guidance, it is specified that preformed metal crowns, placed using the Hall Technique, are the treatment of choice for managing lesions that require intervention but no pulpal therapy [5].

Non-restorative cavity control, is “the approach to make the cavitated caries lesions accessible to tooth cleaning by removal of overhanging enamel margins” [24]. This is suggested as an option for management of caries over 1/3 into dentine in primary teeth by SDCEP guidance [5]. There is poor evidence on the suitability of this option and the authors would be reluctant to suggest this other than the rare situation when no other treatment is possible, the child is co-operative for this treatment alone, and excellent oral hygiene and dietary practices are in place at home.

The use of SDF in practice alongside restorative options especially Atraumatic Restorative Technique (ART), have been, referred to as the SMART (Silver Modified ART) or SMART Hall where the Hall Technique is used following SDF application [25]. No guideline discussed this, but it is a recent technique and there is very little evidence apart from case reports and some very recent clinical trial work [26].

Key themes from these guidelines include the move to selective caries removal and avoidance of complete caries removal unless in specific circumstances in anterior teeth only [3]. For “early lesions” in primary and permanent teeth with and without cavitation, several guidelines recommend biological management including site specific prevention and fissure sealants [5, 14, 27].

### Pulp therapy

In the guidelines, pulpotomy was recommended in primary teeth with a carious exposure in some circumstances, with pulpectomy only being recommended in exceptional circumstances for restorable teeth. Interestingly, within the context of the UK, pulp therapy is rarely undertaken in a primary care setting. A recent survey of general dentists in Scotland found that 91% do not offer vital pulp therapy to adult patients due to constraints such as their working contract and costs of materials [28]. Although this survey explored adult treatment it would be unlikely that this group of dentists offers vital pulp treatment to children and not adults, if cost and materials are being cited as barriers. Whilst undergraduate teaching for dentists and therapists in many UK dental schools still include pulp therapy, patients would typically be referred to a clinician with enhanced skills if this approach was required, in accordance with commissioning guidance [3, 29]. As such, there is a need to gain a consensus on whether these recommendations should be taken forward in the development of a paediatric caries curriculum for undergraduate dental and therapy students in the UK, or whether these techniques should instead be taught as an advanced skill at postgraduate level [3, 29].

For permanent teeth with caries into pulp, a partial pulpotomy was recommended in one guideline [12]. This is an evolving area of research with a current randomised control trial underway in the UK to contribute to the evidence base on pulpotomy versus root canal treatment in primary care [28].

Regenerative endodontic treatments were supported by one guideline [12]. This was based on evidence from a position statement by the American Association of Endodontics and a ‘Colleagues for Excellence’ guide, with no precise indications for this option other than immature teeth with pulp necrosis [30, 31]. Most evidence surrounding regenerative endodontics relates to traumatic dental injuries. Although both dental trauma and dental caries, can result in a loss of pulp vitality, the nature of the resulting infection is likely to be different, as may be the prognosis following this procedure.

## Materials

### Amalgam

One US based guideline states that amalgam is not recommended except in some cases when a tooth is anticipated to exfoliate within two years [12] but has limited applicability for UK dental schools working within regulations, such as the EU directive outlawing the use of amalgam in children under 15 except when unavoidable [32]. No guidelines developed within Europe advocate the use of amalgam in CYP. This contrasts with current practice in the UK, shown in findings from the aforementioned evaluation of paediatric caries management teaching practices [3].

### GIC

Glass ionomer cement definitive restorations are advised against by some guidelines [14]. This continues to be a contentious issue, with the type of glass ionomer cement probably the most important factor in its success [33].

### Resin-based materials

Given the restrictions on use of amalgam and the limitations of GIC, there is an increasing reliance on use of resin-based composite materials for definitive restorations. As such, it is unsurprising that these materials were advocated in all included guidelines. This was in particular the AAPD Pediatric Restorative Dentistry and SDCEP Prevention and Management of Dental Caries in Children documents, due to composite’s comparable success to amalgam [5, 14].

### Evidence gaps

Gaps in evidence were identified within the guidelines, for example, on how to manage early cavitated carious lesions of minimal depth which would require complete caries removal solely for the purposes of providing adequate depth for a retentive restoration [1]. These gaps may have been addressed in some of the guidelines we did not include. Nevertheless, they are omitted from otherwise comprehensive and high-quality guidelines.

The variability in terminology, for example, continued use of non-specific terms such as “early lesions” and use of Interim Therapeutic Restoration in US-based documents in place of Atraumatic Restorative Treatment, indicate there is a still no widespread adoption of international consensus on terminology [24].

None of the guidelines recommended tooth tissue removal for early carious lesions, in stark contrast with current teaching practices in the UK [3].

In part, because of inappropriate and inexact use of terminology, none of the guidelines specifically defined carious lesions limited to enamel, how these should be classified and therefore this poses a challenge in selecting the most appropriate treatment option as some clinical judgement is required on accurate diagnosis.

Another challenge not addressed by the guidelines is monitoring caries lesion transition, which is recommended by some guidelines, without specific detail on how [5, 16, 34]. Current record keeping only allows for gross scoring of the presence or absence of carious lesions on a surface, so it is not possible to tell whether lesions have progressed over time. The International Caries Detection and Assessment System (ICDAS) or photographs may help with this but are rarely used and there is no evidence on their accuracy in monitoring progression.

### Context and relevance

This scoping review, undertaken to inform consensus discussions for the development of a UK undergraduate curriculum for caries management in CYP, has identified gaps in guidelines including the classification of early carious lesions and how early cavitated lesions should be managed for CYP. These key findings must be considered in discussions with stakeholders in the UK, with consideration of the findings of preceding work that evaluated the current teaching of caries management in CYP [3]. Areas for exploration in consensus discussions include total integration of biological caries management, selective caries removal and the consideration of whether a pulpotomy for the management of caries is a specialist treatment that requires onward referral.

Furthermore, is important to note that UK dental schools currently provide teaching for students due to graduate and work largely within the National Health Service. There is an expectation that further postgraduate training would be required for delivery of more specialist level procedures. This is in part, related to current UK remuneration systems and possibly the lack of suitable guidelines for incorporation in teaching. As such, students are unlikely to be taught some of the techniques that are mentioned in recommendations in these guidelines, such as use of non-fluoride-based remineralisation agents, resin infiltration for proximal carious lesions, or regenerative endodontic treatments. Further discussion on whether these approaches should be included in a new curriculum would be warranted.

These findings are relevant to those involved in undergraduate teaching of paediatric dentistry, those who develop undergraduate curricula and policymakers.

### Strengths and limitations

Rigorous methodology was used when undertaking this review. This involved blinded screening for eligibility, the assessment of the quality of each guideline using the AGREE II tool and independent review of each guideline by at least two researchers. Meetings were held for agreement and discussing results. Authors of relevant guidelines were also contacted for clarity and to ensure the inclusion of relevant sources. Limitations include the possibility of missed literature in the grey literature search, although every effort was made to find relevant guidelines. There was a lack of high quality, methodologically transparent guidance. Although initially 16 guidelines were eligible for inclusion, assessment of quality using the AGREE II tool meant that only eight guidelines were suitably rigorous to include in the analysis. There were also instances of contradictory recommendations.

## Conclusions

This scoping review identified a limited number of high-quality guidelines suitable for shaping a UK undergraduate dental curriculum in caries management for CYP. However, there were guidelines of sufficient quality for data synthesis generally supportive of biological approaches, which is largely contradictory to current UK undergraduate teaching. There were some gaps in evidence that need to be addressed in future research and guideline development. The evidence synthesised from this review will be used as the basis for deriving a consensus on the content of a new undergraduate curriculum for paediatric caries management.

### Electronic supplementary material

Below is the link to the electronic supplementary material.

Supplementary Material 1

Supplementary Material 2

## Data Availability

Data sharing is not applicable to this article as no datasets were generated or analysed during the current study.
