# Supplementary material for: A scoping review of guidelines on caries management for children and young people to inform UK undergraduate core curriculum development
Source: BMC Oral Health. 2024 Apr 26;24:494. doi: 10.1186/s12903-024-04278-7 (PMC11055302; doi:10.1186/s12903-024-04278-7)
Supplement: Supplementary file 1 — Supplementary Material 1 [file 12903_2024_4278_MOESM1_ESM.docx]

Supplementary Material

**Appendix 1.**

**Search strategy**

Database: MEDLINE via PubMed <01/01/2007 to 25/01/2024>

1. Caries management AND guidelines [tiab:~0]
2. Caries management AND protocol [tiab:~0]
3. Caries management AND guideline [tiab:~0] *

1 and 2

2 and 3

Database: Web of Science <01/01/2007 to 25/01/2024>

1. Caries management AND guidelines [tiab:~0]
2. Caries management AND protocol [tiab:~0]

1 and 2

Database: Cochrane Library <01/01/2007 to 25/01/2024>

1. Caries management AND guidelines
2. Caries management AND protocol

Database: Trip Medical Database <01/01/2007 to 25/01/2024>

1. Caries management AND guidelines
2. Caries management AND guidance
3. Caries management AND protocol

* Denotes search string excluded from future sea
